# Effect of high‐hydrostatic pressure on the digestibility of egg yolk and granule

**DOI:** 10.1111/1750-3841.17051

**Published:** 2024-03-29

**Authors:** Yosra Ben‐Fadhel, Véronique Perreault, Alice Marciniak, Romuald Gaillard, Yves Pouliot, Guillaume Brisson, Alain Doyen

**Affiliations:** ^1^ Department of Food Sciences Université Laval Quebec City Quebec Canada; ^2^ Institute of Nutrition and Functional Foods (INAF) Université Laval Quebec City Quebec Canada; ^3^ Department of Food Sciences University of Guelph Guelph Ontario Canada

**Keywords:** Egg yolk, granule, high hydrostatic pressure, in vitro digestion, degree of hydrolysis, protein profile, phospohlipids

## Abstract

**Abstract:**

The impact of high hydrostatic pressure (HHP) on protein digestibility of egg yolk and egg yolk granule was evaluated by static in vitro digestion using the standardized INFOGEST 2.0 method. The degree of hydrolysis (DH) and the phospholipid content were determined during digestion, and the protein and peptide profiles were characterized by sodium dodecyl sulfate‐polyacrylamide gel electrophoresis and reverse phase‐high pressure liquid chromatography (RP‐HPLC). The results showed that HHP induced protein aggregation in egg yolk and granule, mainly by disulfide bridges, which were not disrupted in the oral phase. Proteolysis during the gastric phase improved egg yolk and granule protein solubility, regardless of whether HHP was applied. However, the extent of the samples’ digestibility was not affected, with DH values ranging from 15% to 20%. During the intestinal phase, the DH of egg yolk protein (∼40%) was higher than that of the granule (∼25%), probably due to the denser structure of the granule reducing the accessibility of intestinal enzymes. The DH, peptide, and protein profiles of control and HHP‐treated egg yolk showed similar protein digestion behaviors for both gastric and intestinal phases. Among the different proteins, only the digestibility of β‐phosvitin in HHP‐treated granule was enhanced. Consequently, applying HHP to granules represents an interesting process that improves the digestibility of phosvitin with the potential to generate bioactive phosvitin‐derived phosphopeptides.

**Practical Application:**

High hydrostatic pressure, mainly used as a preservation process, did not impair the nutritional quality of the egg yolk and granule proteins but improved the susceptibility of phosvitin (protein contained in egg yolk) proteolysis to produce bioactive phosphopeptides. Consequently, applying HHP to granules represents an interesting process that improves the digestibility of phosvitin.

## 1 INTRODUCTION

Egg is an integral part of the human diet and is widely used in the food industry due to its nutritional, sensorial, and techno‐functional properties. Notably, egg yolk is used in the formulation of different food products in relation to its gelation and emulsifying properties. Egg yolk is composed of 50% water, 30% lipid, and 16% protein. Specifically, the lipid and protein fractions consist of 68% low‐density lipoproteins (LDL), 16% high‐density lipoproteins (HDL), 10% livetins (glycoproteins), and 4% phosvitin (McCully et al., 1962).

From the egg yolk, two specific fractions, granule and plasma, are recovered after a centrifugation step (10,000 × *g* for 45 min) (Anton, 2007). The soluble plasma fraction is composed of LDLs (85%) and livetin (15%) and represents an interesting source of bioactive components such as immunoglobulin Y and phospholipids (Da Silva et al., 2022). The insoluble granule consists of circular complexes of proteins and lipids with diameters ranging from 0.3 to 2 µm composed of HDLs (70%), phosvitin (16%), and LDLs (12%) (He et al., 2023). HDL is a valuable source of phospholipids (phosphatidylcholine [PC], phosphatidylinositol [PI], phosphatidylethanolamine, lysophosphatidylcholine, and sphingomyelin) and folate, with low cholesterol content. In addition, the granule demonstrates good emulsifying and gelling properties (Valverde et al., 2016). Consequently, granule represents a promising ingredient for the human diet and the formulation of innovative food products by the food industry (Huopalahti et al., 2007; Laca et al., 2015). However, due to its very compact and poorly hydrated structure, different strategies have been applied to destabilize the egg yolk granule to improve its techno‐functional properties.

Typically, increasing the ionic strength of granule solution has improved its solubility and emulsion stability (Anton & Gandemer, 1997). Moreover, it was demonstrated that the application of conventional high‐pressure homogenization (0.3 to 20 MPa) on granule had little effect on its microstructure and techno‐functional properties, whereas the application of ultra‐high pressure homogenization (300 MPa for 1 and 4 passes) improved the water and oil binding capacities of granules (Gaillard et al., 2023). In addition, high hydrostatic pressure (HHP) processing has been used to destabilize the egg yolk granule. It was demonstrated that HHP induced the extraction of phosvitin and folate from granule to plasma fraction by disruption of the phosphocalcic bridges between HDL and phosvitin (Duffuler et al., 2020; Naderi et al., 2017). However, to use HHP‐treated granule as a food ingredient, knowledge of its digestibility is crucial. Consequently, the aim of this study was to investigate the effect of HHP on the digestibility of egg yolk and egg yolk granule by using the harmonized INFOGEST 2.0 static in vitro digestion method. More specifically, the degree of hydrolysis of proteins, as well as protein/peptide profiles, were monitored after the oral, gastric, and intestinal phases. The evolution of the phospholipid content during digestion was also investigated.

## 2 MATERIALS AND METHODS

### 2.1 Preparation of egg yolk granule

Fresh hen eggs were purchased from a local supermarket and stored at 4°C until preparation. Preparation of egg yolk granule was the same as described by Naderi et al. (2014). Briefly, eggs were manually broken and the albumen was discarded. The residual albumen was eliminated from the yolk by absorption on filter paper (Whatman, MA, USA). The vitelline membrane of the yolk was then removed with tweezers. A part of the egg yolk was kept liquid, then diluted in distilled water (1:1) and centrifuged at 10,000 × *g*, 4°C for 45 min to generate the granule, which was freeze‐dried. The second part of the egg yolk was freeze‐dried.

For use as controls, prepared egg yolk and granule were solubilized in distilled water at a protein concentration of 1% (w/v) to produce egg yolk control (EYC) and granule control (GC). For treatment, prepared egg yolk and granule were pressurized at 600 MPa for 10 min at 20°C in a discontinuous hydrostatic pressurization unit (Hiperbaric 135 L, Hiperbaric, Burgos, Spain) to produce HHP‐treated egg yolk (EYP) and granule (GP). In our previous works, these pressurization parameters (600 MPa for 10 min) were demonstrated as the most efficient to induce a drastic destabilization of the granule (Duffuler et al., 2020) and the generation of a granule ingredient with interesting emulsifying properties (Giarratano et al., 2020). During pressurization experiment, the temperature of the pressure transmitting fluid (water) was maintained at 20°C. Due to the low volume of granule (∼100 mL) compared to the large volume of water (135 L) used during pressurization, the increase of granule temperature due to adiabatic compression was estimated as very low with no impact on granule structure. All samples were freeze‐dried and characterized until static in vitro digestion experiments.

**FIGURE 1:**
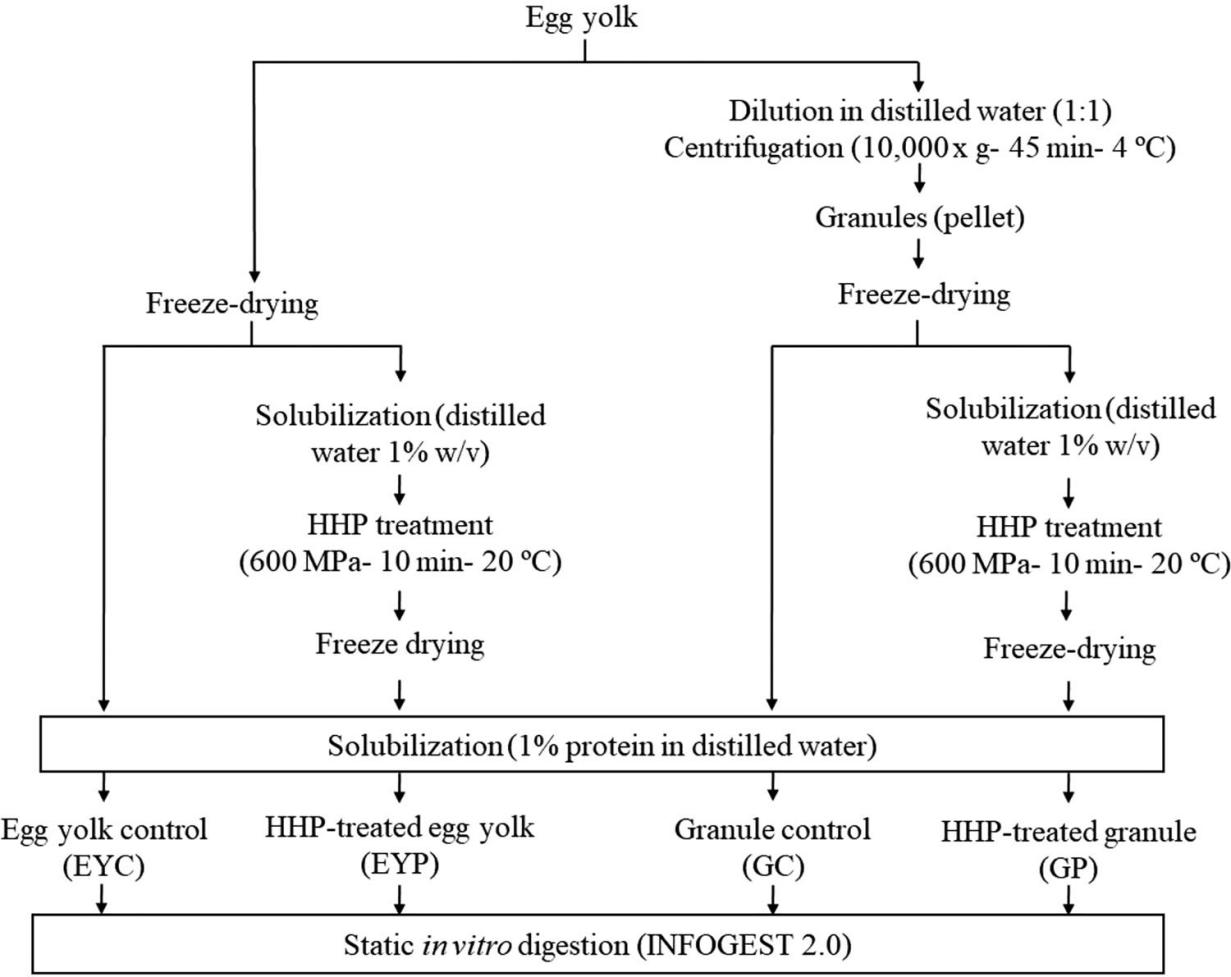
Flowchart of the production of control and high hydrostatic pressure‐treated egg yolk and granule fractions.

The dry matter and ash content determinations were carried out according to the Association of Official Agricultural Chemists (AOAC) 923.03 (dry matter) and 925.09 (ash) methods. The crude protein content was obtained by the Dumas method (LECO FP‐528, Model 601‐500, LECO, St. Joseph, MI, USA) using a protein‐to‐nitrogen conversion factor of 6.25 (Gaillard et al., 2023) and the lipid content was determined by the Mojonnier method (AOAC International 925.32). The proximate composition of the control and EPYs and granules is presented in Table 1. The flowchart of the production of egg yolk and granule fractions is presented in Figure 1.

**TABLE 1:** Proximate composition of control and high hydrostatic pressure‐treated egg yolk and granule expressed on a dry basis.

Fractions	Total solids	Proteins	Lipids	Minerals
	% (w/w)			
EYC	99.25 ± 0.41 ^a^	32.77 ± 0.29 ^a^	54.04 ± 3.47 ^b^	3.34 ± 0.20 ^a^
GC	99.02 ± 0.76 ^a^	60.56 ± 1.09 ^b^	36.13 ± 6.19 ^a^	6.37 ± 0.46 ^b^
EYP	98.71 ± 0.22 ^a^	33.95 ± 1.33 ^a^	54.15 ± 3.56 ^b^	3.69 ± 0.17 ^a^
GP	99.46 ± 0.04 ^a^	60.49 ± 0.21 ^b^	33.66 ± 3.44 ^a^	6.94 ± 0.19 ^b^

*Note*: Results expressed as mean ± standard deviation. Data with different letters within each column are significantly different at *p *≤ 0.05 (Tukey test, *α* = 0.05, *n* = 3).

Abbreviations: GC, granule control; GP, HHP‐treated granule; EYC, egg yolk control; EYP, HHP‐treated egg yolk.

### 2.2 In vitro static digestion

Static in vitro digestion of egg yolk and granule fractions was performed in triplicate using the standardized INFOGEST 2.0 method (Brodkorb et al., 2019). Briefly, suspensions (1% protein w/w) of control and HHP‐treated yolks and granules were prepared in distilled water. An enzyme blank consisting of a digestion tube with all enzymes and simulated fluids but without egg yolk and granule was also prepared. The in vitro static digestion, which consisted of oral, gastric, and intestinal phases, was performed at 37°C under agitation at 200 rpm using a shaking device. For the oral phase, control and HHP‐treated yolk and granule samples were mixed with simulated salivary fluid (1:1 w/w), and the mixtures were incubated at pH 7.0 for 2 min. After 2 min of oral digestion, samples were collected, aliquoted, and frozen. The remaining mixtures were diluted (1:1 w/w) with the simulated gastric fluid (the final activities of pepsin and lipase were 2000 and 60 UI/g, respectively) and incubated under agitation at pH 3.0 for 2 h. During incubation, egg yolk and granule samples were collected every 30 min and Pepstatin A (concentration of 1 µM) was added to stop the gastric digestion. The gastric chyme was then diluted with simulated intestinal fluid (1:1 w/w) containing bile (10 mM) and pancreatin (100 UI/g) and incubated at pH 7 for 2 h. During the intestinal phase, egg yolk and granule samples were collected every 30 min. Pefabloc SC (concentration of 5 mM) was added to both collected samples and samples recovered at the end of incubation to stop intestinal digestion. All samples recovered during and at the end of digestion were stored at −80°C until analysis.

### 2.3 Degree of hydrolysis

The degree of hydrolysis (DH) of control and EPY and granule fractions as well as samples recovered during in vitro digestion experiments, including the enzyme blanks, was calculated from the measurement of the primary amines by o‐phthaldialdehyde (OPA) assay according to Church et al. (1983). Due to the non‐optimal solubility of the control and EPYs and granules before and during the oral phase (0 and 2 min of digestion), only samples recovered during and at the end of gastric and intestinal digestion phases were analyzed. First, control and EPYs and granules as well as their digesta generated from the gastric and intestinal phases were diluted in NaCl solution (0.5 M), sonicated for 30 min, and then shaken overnight at 4°C to improve solubility. Solutions were then centrifuged at 2000 × *g* for 5 min to remove the insoluble particles. The absorbance was measured at 340 nm by spectrophotometry (HP 8453 spectrophotometer, Agilent Technologies, Inc. Santa Clara, CA, USA). A standard curve was prepared using DL‐Leucine from 0.75 to 3 mM. The DH was calculated according to the following equation:

(1) $$DH(\%)=\left[\frac{NH_{2}\mathrm{sample}(t)-NH_{2}\mathrm{blank}(t)}{NH_{2}\left(\mathrm{tot}\right)}\right]\times 100$$

where NH_2_(*t*) was the concentration of primary amines after *t* min of digestion and NH_2_(tot) was the concentration of the total primary amines measured after total acid hydrolysis (Su et al., 2021). All measurements were carried out in duplicate for each digesta.

### 2.4 Protein profiles

Protein profiles of samples collected before and during static in vitro digestion were obtained by native and sodium dodecyl sulfate‐polyacrylamide gel electrophoresis (SDS‐PAGE) under reducing and non‐reducing conditions (Duffuler et al., 2020). First, all samples were sonicated for 30 min and shaken overnight at 4°C to maximize their solubilization. Next, samples were mixed with an equal volume of either native, Laemmli reduced (with β‐mercaptoethanol) or non‐reduced (without β‐mercaptoethanol) loading buffer. Samples in the reduced group were boiled for 5 min. The loading volumes were 7 µL for samples and 5 µL for molecular weight markers (Precision Plus Protein^™^ 161–0373 All Blue Prestained Protein Standards, Bio‐Rad, Mississauga, ON, Canada). Electrophoresis was performed using 4%–20% TGX stain‐free polyacrylamide gel (Bio‐Rad) and run at a constant current of 15 mA/ gel for approximately 70 min at room temperature. Proteins were visualized using Coomassie brilliant blue staining containing 0.01 M aluminum nitrate for 1 h and destained with a solution of methanol, acetic acid, and distilled water (1:1:8 v:v:v). The gels were digitized using Chemidoc MP (Bio‐Rad). Proteins were identified according to the published work of Duffuler et al. (2020).

### 2.5 Reversed‐phase high‐performance liquid chromatography

The protein and peptide profiles of control and EPY and granule fractions as well as samples collected during and at the end of static in vitro digestion were obtained by RP‐HPLC analyses using an Agilent system (series 1100, Agilent Technologies) equipped with a variable UV visible detector operating at 214 nm (series 1100, Agilent Technologies) (Pouliot et al., 2009). All samples were diluted in NaCl solution (0.5 M), sonicated for 30 min, shaken overnight at 4°C to improve solubility and centrifuged at 2000 × *g* for 5 min. Pellets were discarded and supernatants were recovered for analysis. Protein and peptides were analyzed with a Luna 5 µm C18 column (2 i.d. 250 mm, Phenomenex, Torrance, CA) at a flow rate of 0.2 mL/min at 45°C. Twenty microliters of the sample was injected and eluted with a linear gradient of mobile phase B (0.1% v/v trifluoroacetic acid (TFA) in acetonitrile) and mobile phase A (0.1% v/v TFA in Milli‐Q water) starting from 2% to 50% of B in 50 min, then 100% B from 70 to 90 min, and then drastically decreased to 2% and was maintained up to 116 min. Data acquisition and chromatography analyses were performed using Chem Station software version B.01.03 (Agilent Technologies).

### 2.6 Phospholipid determination

The Stewart assay was used for the determination of phospholipid content in control, EPY, and granule fractions as well as in samples collected during and at the end of each digestion step. Phospholipids were extracted by mixing 300 µL of yolk, granule, and digesta samples with 900 µL of a mixture of CHCl_3_ and MeOH at a ratio of 2:1. The solutions were vigorously vortexed for 1 min and centrifuged at 10,000 × *g* for 10 min at 4°C. The organic phase (bottom), mainly composed of phospholipid, was recovered and a volume of 50 µL was mixed with 1950 µL of CHCl_3_ and 2 mL of ammonium ferrothiocyanate. All samples were stirred for 1 min and centrifuged again at 2000 × *g* at 4°C for 5 min. The aqueous phase was discarded, the organic phase recovered, and its absorbance measured in a quartz cuvette (𝜆 = 485 nm). A blank was prepared with 50 µL of CHCl_3_ and subtracted from the sample value. A standard curve was prepared with phosphatidylcholine (0 to 1 mg/mL) from hen egg yolk (P3556, Sigma, St. Louis, MO, USA).

### 2.7 Statistical analysis

Statistical analyses were performed using PASW Statistics 18 software (IBM Corporation, Somers, NY, USA). All experiments were performed in triplicate. Phospholipid content and DH were subjected to a one‐way analysis of variance (ANOVA). More specifically, for DH, values calculated for each digestion time and conditions (control and HHP‐treated granules and egg yolk) were compared. The differences of the means between the samples were determined using the Tukey test and a *p*‐value < 0.05 was considered statistically significant.

## 3 RESULTS

### 3.1 Degree of hydrolysis

**FIGURE 2:**
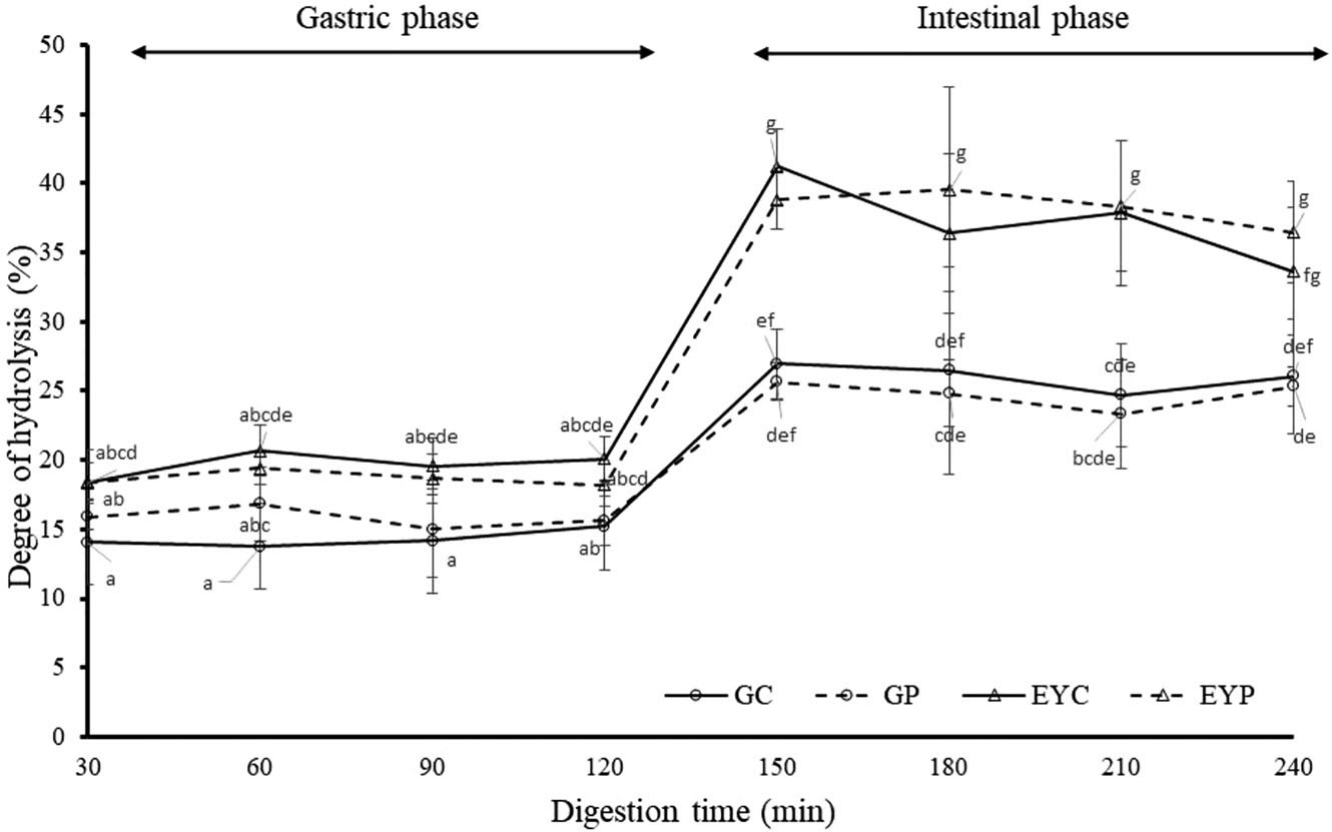
Degree of hydrolysis of control and high hydrostatic pressure (HHP)‐treated egg yolks and granule during gastric and intestinal phases. Values are mean ± standard deviation of duplicate measurements of three repetitions. All DH values calculated at each digestion time and for all conditions (GC, GP, EYC, and EYP) were compared. Different letters indicate significant differences (Tukey test, *α* = 0.05, *n* = 3). GC, granule control; GP, HHP‐treated granule; EYC, egg yolk control; EYP, HHP‐treated egg yolk.

The DH values of control and EPYs and granules, as well as their digesta, are presented in Figure 2.

During gastric phase, from 30 to 120 min, the application of HHP on egg yolk (EYP) and granule (GP) had no impact on proteolysis since similar and constant DH values (*p* > 0.05), ranging between 15% and 20%, were obtained for all conditions (GC, GP, EYC, and EYP).

At the beginning of the intestinal phase (150 min), the DH of egg yolk and granule samples increased significantly (*p* ≤ 0.05) compared to the gastric phase (∼25% and ∼40% for the granule and egg yolk samples, respectively). However, as mentioned for gastric digestion, no impact of the application of HHP was noticed since the DH values remained stable throughout the intestinal phase for egg yolk (EYC and EP) and granule (GC and GP) samples (*p* > 0.05). Nevertheless, differences were noticed between egg yolk and granule samples. Indeed, the DH was higher for egg yolk samples (EYC and EYP) compared to that of granule samples (GC and GP) at 150, 180, and 210 min. This tendency was also observed at 240 min except that similar DH values were calculated for control egg yolk and granule samples (*p* > 0.05).

### 3.2 Protein and peptide profiles of egg yolk and granule fractions by native, non‐reducing, and reducing gel electrophoresis

**FIGURE 3:**
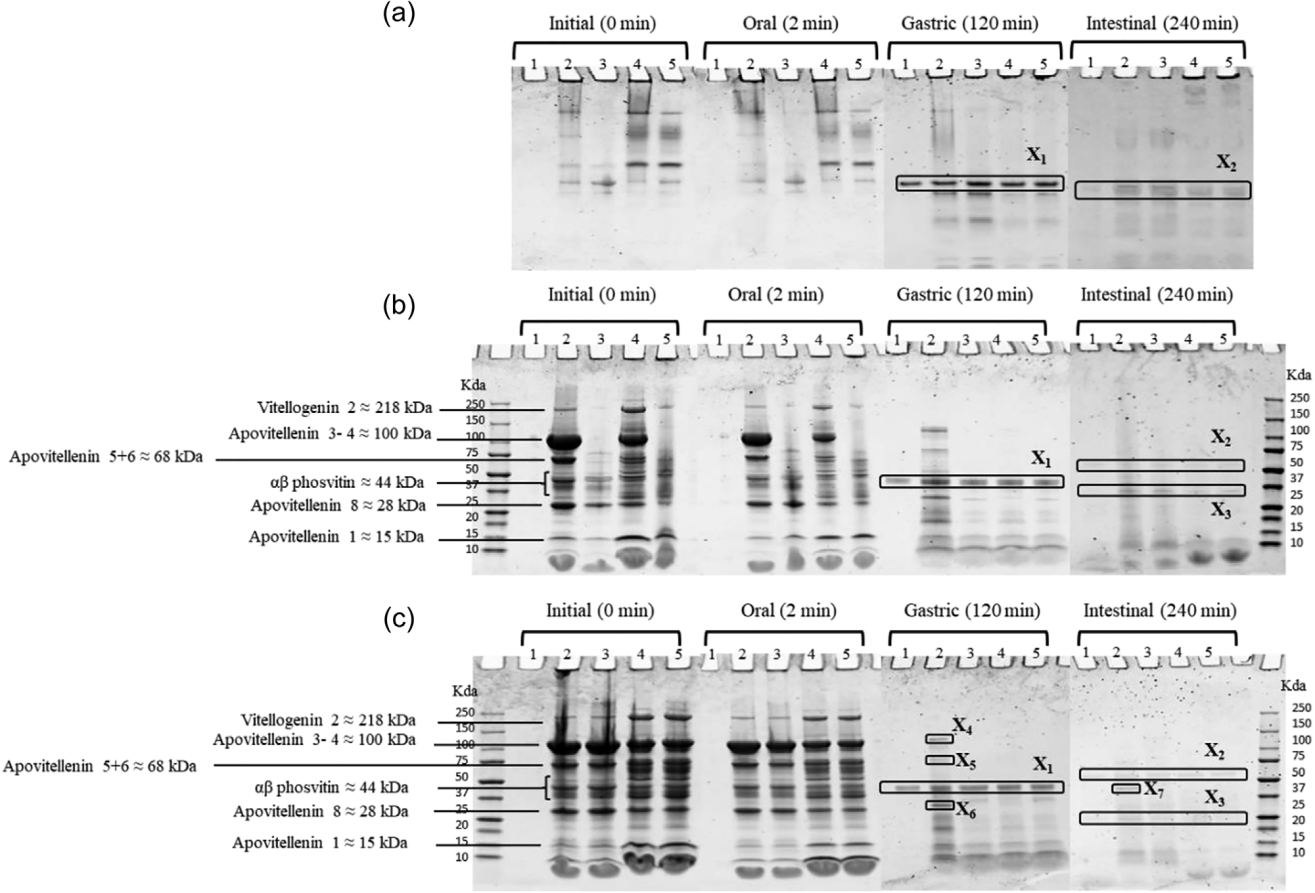
Protein profile of blanks (well #1), granule control (GC, well #2), high hydrostatic pressure (HHP)‐treated granule (GP, well #3), egg yolk control (EYC, well #4), and HHP‐treated egg yolk (EYP, well #5) during digestion by gel electrophoresis under native (a), denatured non‐reduced (b), and denatured reduced (c) conditions. X_1_ to X_3_ represents gastric (X_1_) and intestinal (X_2_ and X_3_) enzymes used during digestion. X_4_, X_5_, X_6_, and X_7_ correspond to APO3‐4, APO5‐6, APO8, and PHO, respectively.

Figure 3 presents the protein and peptide profiles of control and EPYs and granules and their digesta. Native PAGE (Figure 3a) of the initial (before digestion) and oral samples resulted in similar protein patterns. High molecular weight and/or aggregated proteins were detected in wells for all samples before and after the oral phase. The protein patterns of the control and HHP‐treated samples differed with the presence of more high molecular weight bands for the egg yolk than for the granule. In the gastric and intestinal phases, enzyme bands X_1_ and X_2_ (well #1), used during digestion, were the most intense. Low molecular weight proteins and/or peptides were detected after the gastric phase and aggregates initially observed in wells were not detected anymore. After the intestinal phase, the number of bands and their respective intensities decreased compared to the oral and gastric phases. Under non‐reducing conditions (Figure 3b), seven major protein bands corresponding to vitellogenin 2 (VIT2), apovitellenin 3‐4 (APO3‐4), apovitellenin 5–6 (APO5‐6), α‐ and β‐phosvitin (PHOS), apovitellenin 8 (APO8), and apovitellenin 1 (APO1) were identified. For the initial and oral phases, similar protein patterns were obtained for the control egg yolk and granule. However, the intensities of the bands corresponding to APO3‐4, APO5‐6, APO8, and PHOS were higher for GC, and VIT2 and APO1 were higher intensity bands for EYC. High molecular weight proteins were also detected in the wells. The application of HHP largely modified the protein pattern of egg yolk and granule. Indeed, the intensities of VIT2, PHOS, and APO8 decreased drastically, and APO3‐4 (∼100 kDa) and APO5‐6 were not visible for HHP‐treated samples. As the digestion progressed, only bands with molecular weight <37 kDa remained visible and no aggregates were detected in the wells. Control and EPY had similar peptide and protein profiles. However, bands corresponding to APO3‐4 and APO5‐6 of GC that were observed in the GC sample during gastric phase (well #2) were not visible for GP. As expected, bands corresponding to enzymes used during digestion (pepsin and lipase X_1_ ∼ 40 kDa) and intestinal enzymes (X_2_ ∼ 52 kDa and X_3_ ∼ 25 kDa) were observed. Under reducing conditions (Figure 3c), the protein profiles of control and EPY and granule were similar for the initial and oral phases and no protein aggregates were detected in the wells. However, a band with molecular weight close to 50–60 kDa was only observed for egg yolk (control and HHP treated). In addition, the band intensities of VIT2 and APO5‐6 were higher for egg yolk while APO3‐4 intensity was lower. As indicated for the non‐reducing condition, the number of bands and their intensities decreased drastically in the gastric and intestinal phases, and mainly the bands corresponding to enzyme (X_1_, X_2_, and X_3_) were detected. HHP treatment had no effect on the egg yolk protein pattern during any digestion step, showing the disappearance of almost all bands with the gastric phase regardless of the application of HHP (Figure 3c). However, HHP applied to granule samples resulted in better digestibility of APO3‐4 (X_4_), APO5‐6 (X_5_), and APO8 (X_6_) during gastric phase. HHP treatment has also induced better digestibility of PHO (X_7_) in granules during the intestinal phase.

### 3.3 Reversed‐phase high‐performance liquid chromatography

**FIGURE 4:**
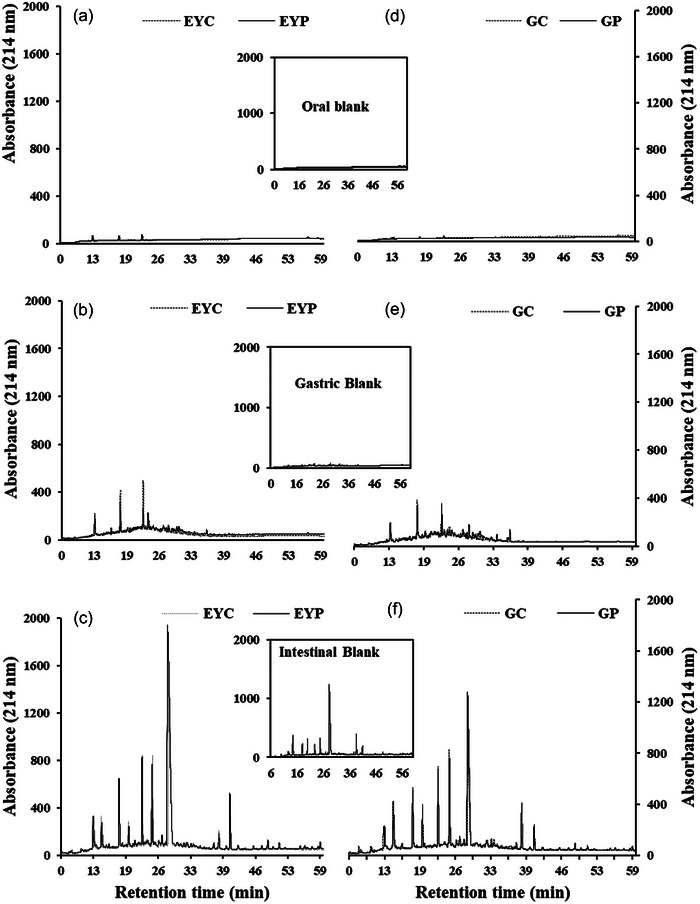
RP‐HPLC peptide profile of control (EYC) and high hydrostatic pressure (HHP)‐treated (EYP) egg yolk (a–c) as well as control (GC) and HHP‐treated granule (GP) (d–f) during oral (top part of figure), gastric (middle part of figure), and intestinal (bottom part of figure) digestion phases. The box indicated in each chromatogram corresponds to the enzyme blank for the different phases of digestion.

The protein and peptide profiles of control and EPY and granule as well as digesta are presented in Figure 4. Three peaks with very low intensities were detected only for control and EPY during the oral phase at retention times of 13, 18, and 23 min (Figure 4a). During the gastric phase (Figure 4b), the number and intensity of peaks increased for both egg yolk and granule, regardless of the application of HHP. The main peaks detected had retention times of 13, 18, 23, and 24 min for egg yolk and 13, 18, 23, 28, and 36 min for granule. Moreover, the number of peaks was higher for the granule compared to the egg yolk. For both egg yolk and granule, HHP treatment reduced the intensities of the peaks eluted at 13, 18, and 23 min, whereas it increased the intensities of those eluted between 29 and 36 min. During the intestinal phase (Figure 4c), many low‐intensity peaks were detected for both egg yolk and granule samples, whereas the main peaks corresponded to intestinal enzymes. Moreover, no effect of HHP was noticed for both egg yolk and granule.

### 3.4 Phospholipid content

**FIGURE 5:**
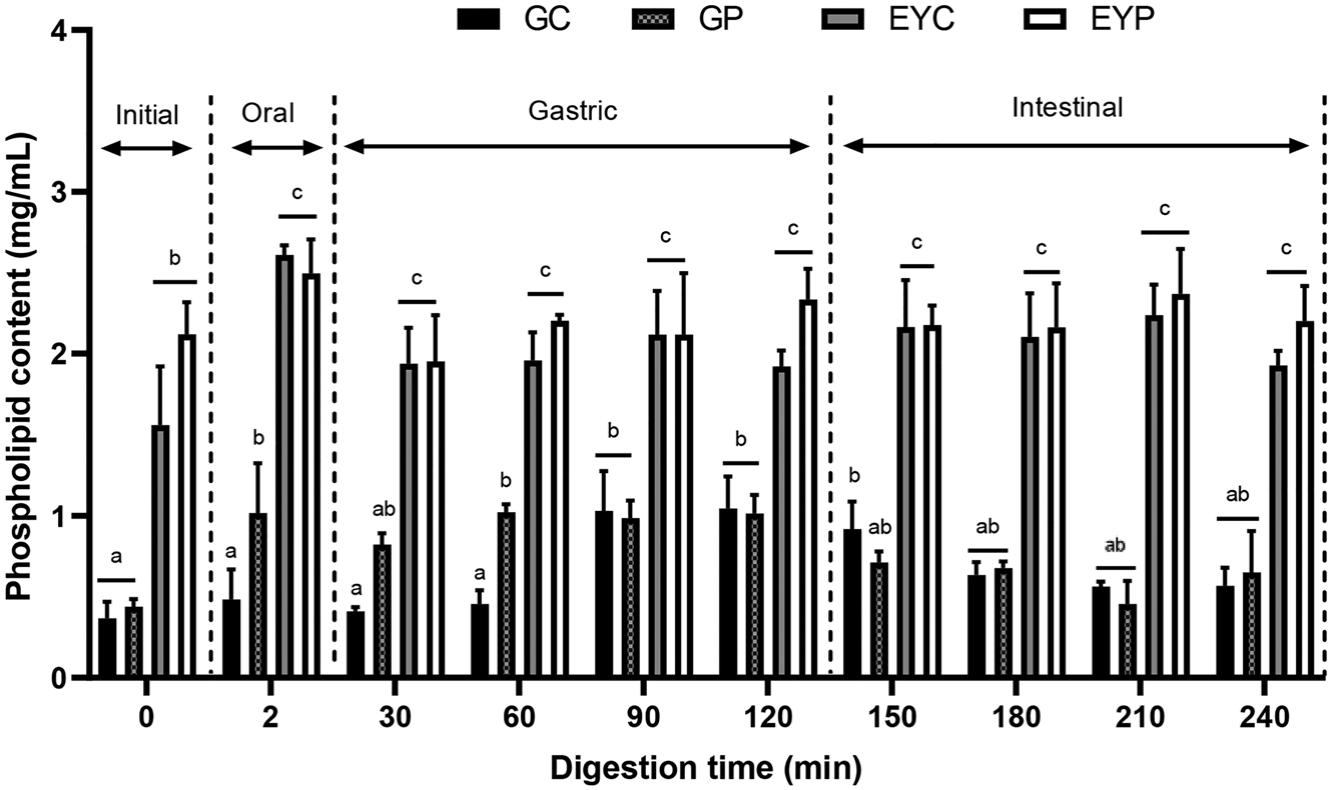
Phospholipid content of control and high hydrostatic pressure‐treated egg yolk and granule before digestion (0 min), and their respective digesta during the oral, gastric, and intestinal phases. Values are mean ± standard deviation of duplicate measurements of three repetitions. Different letters indicate significant differences (Tukey test, *α* = 0.05, *n* = 3).

Figure 5 shows the phospholipid content of control and EPY and granule samples during initial, oral, gastric, and intestinal phases of digestion. Before digestion (*t* = 0 min) and during all digestion phases, the phospholipid content of egg yolk was consistently higher than that of granule. More specifically, the phospholipid content was 1.8, 2.6, 1.9, and 2.2 mg/mL for egg yolk and 0.4, 0.8, 0.9, and 0.7 mg/mL for granule at initial, oral, gastric, and intestinal phases, respectively. In addition, phospholipid content increased during the oral phase to reach 2.6 and 0.8 mg/mL for egg yolk and granule, respectively, and then remained stable during the gastric and intestinal phases. Overall, HHP treatment had no impact on phospholipid content, regardless of the sample (egg yolk and granule) (*p* > 0.05). However, surprisingly, the phospholipid content in the granule increased during the oral phase and after 60 min of gastric digestion after HHP treatment (*p* < 0.05) whereas it remained similar until the end of gastric digestion and during intestinal phase (*p* > 0.05). A similar trend was observed after 30 min of gastric digestion despite the absence of statistical difference.

## 4 DISCUSSION

Previous work has shown that applying HHP to various protein sources improved their digestibility (Cepero‐Betancourt et al., 2020; Linsberger‐Martin et al., 2013; Zhang et al., 2019) due to protein denaturation resulting from the impact of pressurization on protein structure (Deng et al., 2015; Sazonova et al., 2019). It was demonstrated that HHP caused a major destabilization of the egg yolk and granule granular network through the breakage of phosphocalcic bonds leading to the release of phosvitin and folate. Moreover, this destabilization induced the transfer of β‐phosvitin from the granule to the plasma fraction (Duffuler et al., 2020). In this context, structural modifications due to HHP treatment of the egg yolk and granule at 600 MPa for 10 min could improve protein digestibility. As expected, before in vitro digestion, HHP had no impact on the proximate composition of either egg yolk or granule samples (Table 1). During in vitro digestion, the proteolysis of egg yolk was increased compared to granule during the intestinal phase, due to mainly the granule having a denser protein core that slowed enzyme diffusion and proteolytic action (Figure 2) (Bao et al., 2017; Eckert et al., 2019; Orcajo et al., 2013). However, the application of HHP seemed to have no or very little impact on granule and egg yolk digestibility since DH during gastric and intestinal phases were similar, regardless of the application of HHP or not. To validate this observation, the effects of HHP and the peptide profiles of yolk and granule protein during digestion were studied.

Globally, the decrease in protein band intensities as a function of digestion stage (gastric and intestinal digestion) (Figure 3) due to their proteolysis by gastric and digestive enzymes is correlated with the increase of peptide peaks intensities and area under the curve generated by digestion (Figure 4). Interestingly, in the native and non‐reduced gels, the initial and oral phase samples showed a decreased number of bands with HHP for both granule and egg yolk, whereas no effect was observed in the reduced gel (Figure 3). These observations suggested that application of HHP treatment decreased the solubility of egg yolk and granule via aggregation of the proteins, mainly from the LDL, through disulfide bonds (covalent interaction). These observations are consistent with the analysis of the RP‐HPLC protein and peptide profiles (Figure 4), showing the elution of three small peaks which probably corresponded to soluble yolk proteins such as α‐livetin, β‐livetin, and γ‐livetin (Guha et al., 2019). As expected, following the gastric and intestinal phases, a general decrease in band intensity was observed (Figure 3), mainly attributed to both the dilution factor and enzymatic hydrolysis. These results were in line with the increase in DH values (Figure 2) and the increase in the intensity of peaks eluted in the RP‐HPLC profiles (Figure 4b,c). Indeed, as the acidic gastric conditions caused the dissociation and complete solubilization of the aggregated proteins (Causeret et al., 1991), the accessibility of lipase and pepsin to their substrates was improved. The fact that HHP did not impact the peptide and protein profiles of egg yolk within the gastric and intestinal phases suggested complete digestion of the egg yolk proteins, supporting the higher DH values obtained for egg yolk (Figure 2). However, some proteins, mainly apovitellenin (X_4_, X_5_, and X_6_) during the gastric phase and phosvitin (X_7_) during the intestinal phase, were observed in the granule control (Figure 3), suggesting a higher resistance to proteolytic digestion compared to the HHP‐treated granule. Indeed, phosvitin is well known to resist proteolysis by digestive enzymes (protease and trypsin), thermal denaturation, and heat gelation (Anton, 2013; Ishikawa et al., 2007). Our results suggested that phosvitin from HHP‐treated granule was completely digested as shown by the disappearance of its band (X7) in reduced gels (Figure 3c) which probably induced the generation of phosphopeptides, as demonstrated in previous work (Jiang & Mine, 2000, 2001; Ren et al., 2015). The sensitivity of phosvitin to HHP was also reported by Yoo et al. (2017) and by Farjami et al. (2021) and was attributed to the extraction of phosvitin from granule via the breaking of phosphocalcic bridges which makes phosvitin less resistant to enzymes action and therefore easier to digest.

The initial content of phospholipids in egg yolk was three to four times higher compared to the granule. This difference was also reported in previous work (Anton & Gandemer, 1997; Jin et al., 2013). However, only a 1.6‐fold higher phospholipid concentration was observed in egg yolk (∼17%) compared to granule (∼11%) (Figure 5). This difference could be explained by the lower temperature used during the centrifugation step of egg yolk since lipid extraction yield is improved with increased temperature (Price et al., 2018; Sivaramakrishnan & Incharoensakdi, 2019). Globally, literature shows relative high resistance of lipid complexes such as LDLs and HDLs toward high pressure (Golub et al., 2017; Lehofer et al., 2018; Speroni et al., 2005) validating the absence of difference in PL content for the initial stage. Moreover, the stable concentration of phospholipids during gastric and intestinal phases for control and EPY and granule could be related to the resistance of phosphatidylethanolamine and phosphatidylcholine, the main egg yolk phospholipids, to digestion (Mrsny et al., 1986). Moreover, the application of HHP could have enabled phospholipids to form lipid bilayers and micelles which would explain the improved resistance to digestive enzymes observed, since lipolysis is an interfacial reaction (Mat et al., 2016). This potential formation of lipid bilayers and micelles could explain the higher phospholipid contents obtained at oral phase and after 60 min of gastric digestion for HHP‐treated granule compared to the control condition due to a higher phospholipid extraction efficiency (Figure 5).

## 5 CONCLUSION

The application of HHP treatment had no impact on the egg yolk protein profile and digestibility. The same tendency was observed for the granule fraction, except that only α‐ and β‐phosvitin were impacted by pressurization. These new insights demonstrated that HHP, mainly used as a preservation process, did not impair the nutritional quality of the proteins but improved the susceptibility of phosvitin proteolysis to produce bioactive phosphopeptides in the granule fraction.

## AUTHOR CONTRIBUTIONS

**Yosra Ben Fadhel**: Conceptualization; formal analysis; data curation; writing—original draft; writing—review and editing. **Véronique Perreault**: Formal analysis. **Alice Marciniak**: Writing—review and editing. **Romuald Gaillard**: Formal analysis. **Yves Pouliot**: Conceptualization; writing—original draft. **Guillaume Brisson**: Conceptualization; supervision; writing—original draft. **Alain Doyen**: Conceptualization; resources; supervision; writing—review and editing.

## CONFLICT OF INTEREST STATEMENT

The authors declare that they have no known competing financial interests or personal relationships that could have appeared to influence the work reported in this paper.
